# Epidemiological study of hepatitis B and hepatitis C infections in Northeastern China and the beneficial effect of the vaccination strategy for hepatitis B: a cross-sectional study

**DOI:** 10.1186/s12889-018-5984-6

**Published:** 2018-09-03

**Authors:** Shishen Wang, Yuhui Tao, Yuchun Tao, Jing Jiang, Li Yan, Chong Wang, Yaxuan Ding, Jianxing Yu, Dinghui Zhao, Xiumei Chi, Xiaomei Wang, Ruihong Wu, Xiuzhu Gao, Ying Shi, Yazhe Guan, Yingchun Li, Yanli Xing, Haiyan Sun, Changhua Ta, Chao Wang, Junqi Niu, Jing Meng, Hongqin Xu

**Affiliations:** 1 Changchun Center for Disease Control and Prevention, Changchun, 130033 Jilin China; 2 Epidemiology and Statistics, School of Public Health, Jilin University, Changchun, 130021 Jilin China; 3 Department of Clinical Epidemiology, The First Hospital of Jilin University, 130021, Changchun, Jilin China; 4 Department of Hepatology, The First Hospital of Jilin University, No.71 Xinmin Str., Changchun, 130021 Jilin China; 5 Yushu Center for Disease Control and Prevention, Yushu county, Jilin China; 6 Nongan Center for Disease Control and Prevention, Nongan county, Jilin China; 7 Shuangyang Center for Disease Control and Prevention, Shuangyang county, Jilin China; 8 Erdao Center for Disease Control and Prevention, Erdao district, Changchun, Jilin China; 9 Lvyuan Center for Disease Control and Prevention, Lvyuan district, Changchun, Jilin China

**Keywords:** Hepatitis B virus, Hepatitis B vaccination, Hepatitis C virus, Epidemiology

## Abstract

**Background:**

Viral hepatitis, mainly hepatitis B and C, is a serious public health problem worldwide. In China, the prevalence of hepatitis B virus (HBV) infection remains high, while that of hepatitis C virus (HCV) infection is controversial. This study investigated the epidemiology of HBV and HCV infections and assessed the beneficial effect of the vaccination strategy for hepatitis B in Northeastern China.

**Methods:**

From June 2016 to August 2016, 6541 residents of Changchun in Northeastern China were recruited for this cross-sectional study. Demographic characteristics as well as HBV and HCV serological test results were reviewed and analyzed.

**Results:**

Among all study participants, 3.8% and 0.36% tested positive for hepatitis B surface antigen (HBsAg) and anti-HCV, respectively. The HBsAg- and anti-HCV-positive rates were significantly higher in male participants (4.58% and 0.43%) than in female individuals (3.0% and 0.33%). Notably, among all age groups, the lowest rate of HBsAg positivity (0.2%) was found in children born after the implementation of the vaccination strategy for hepatitis B. Conversely, participants aged 40–49 years had significantly greater positive rates of HBsAg (5.9%) compared with those of other age groups. Furthermore, the highest rates of anti-HCV positivity (1.1%) were observed in participants aged 50–59 years.

**Conclusions:**

The rate of HBsAg-positivity declined significantly following successful implementation of the policy on hepatitis B vaccination, indicating a beneficial impact on the control of HBV infection. However, only a slight decrease was observed in the anti-HCV–positivity rate, identifying an area in need of improvement within viral hepatitis prevention and control programs in China.

## Background

Chronic viral hepatitis, primary caused by hepatitis B virus (HBV) and hepatitis C virus (HCV), poses a serious public health problem worldwide. The Polaris Observatory Collaborators estimated that there were approximately 292 million HBV infections in 2016 globally. Of these infections, around 29 million (10%) were diagnosed, and only 4.8 million (5%) of the approximately 94 million individual who were eligible for treatment actually received antiviral therapy [1]. HBV infection is a major cause of chronic liver diseases, such as liver fibrosis, cirrhosis, and even hepatocellular carcinoma (HCC). In fact, HBV infection is responsible for approximately 45% of HCC cases and 30% of liver cirrhosis cases [2]. The recent Global Burden of Disease study reported that HBV infection is the 10th leading cause of death globally, with an estimated 786,000 deaths attributed to HBV each year [3]. The prevalence of HBV infection varies greatly among different regions of the world. In China, its incidence remains particularly high, with two viral hepatitis serological surveys conducted in China in 1992 and 2006 reporting, respectively, that 9.8% and 7.2% of the nationwide population tested positive for hepatitis B surface antigen (HBsAg) [4].

In contrast to HBV infection, HCV infection affects almost every country in the world [5–7]. Approximately 80 million individuals are estimated to have chronic HCV infection, which corresponds to a global prevalence of 1.1%. Annually, an estimated 700,000 persons with chronic HCV infection die without receiving treatment [8].China was once considered a high endemic-area for HCV infection [9], with the 1992 national epidemiological survey finding that 3.2% of the general population tested positive for anti-HCV, with blood or blood product transfusions identified as a major route of HCV infection [10]. However, the 2006 survey found that the prevalence markedly declined from 3.2 to 0.4% [11]. Since 2014, new oral direct-acting antivirals (DAAs) have transformed HCV treatment, making treatment safer and simpler. In countries of the World Health Organization (WHO) Western Pacific Region, an estimated 211,100 people were treated with DAAs by September 2016, and these included 200,100 patients in China.

As HBV and HCV are highly contagious and transmitted through blood transfusions as well as sexual and vertical (perinatal transmission) routes [12–14], improvements in the awareness of the viral infection routes among the general population are expected to reduce the risks of HBV and HCV infections. In China, a policy of free vaccination against hepatitis B for newborn infants and children was implemented in 2002. In this prospective study, we investigated the epidemiology of HBV and HCV infections using a questionnaire that collected data on awareness of hepatitis virus infection as well as the results of laboratory tests for HBV and HCV infections and evaluated the beneficial effect of the vaccination strategy for hepatitis B in Northeastern China.

## Methods

### Participants

Using the expected HBsAg prevalence for different age groups in the study (0.3% for age 1–4 years, 3% for age 5–14 years, 4% for age 15–29 years, 9% for age 30–59 years), a level of confidence of 95%, power of 80%, and tolerance error ranging from 20 to 75% (75% for age 1–4 years, 25% for age 5–14 years, 25% for age 15–29 years, 20% for age 30–59 years), estimating a response rate of 80%, the desired sample size was 6518, and an additional 1613 Children < 5 years, 1412 children 5–14 years, 1863 participants 15–29 years and 1227, adults 30–59 years. A total of 6541 individuals in Changchun City of Northeastern China were enrolled in this cross-sectional study from June 2016 to August 2016. The target study sample was randomly chosen from residents who were aged < 60 years and had lived in the city for at least 6 months using multistage stratified cluster random sampling. Briefly, in the first stage, probability proportional to size (PPS) sampling was used to randomly choose clusters of 15 districts/counties from each region. In the second stage, one study individual was randomly chosen from each household in the participating communities.

Prior to enrollment, written informed consent was obtained from each participant or their guardian, if the patient was a minor aged < 16 years. The present study was reviewed and approved by the Ethics Committee of the Changchun Center for Disease Control and Prevention.

### Data collection

A questionnaire was designed to collect information on the demographic characteristics, history of receiving the hepatitis B vaccine, surgical procedures, blood transfusion, traumatic or cosmetic operations, acupuncture, teeth cleaning, and HBV and HCV infection in family members. All investigation forms were completed by physicians through face-to-face interviews. For children aged < 16 years, the information was provided by a parent or legal guardian.

### Serological testing

For serological examinations, blood samples were collected without anticoagulant from each participant (5 mL from the individuals aged > 2 years and 2 mL from those aged < 2 years). The samples were subsequently separated by centrifugation at 1800×g for 10 min at room temperature (RT) and stored at − 80 °C. The procedures for sample collection and storage were performed as soon as possible and no longer than 6 h according to the standard protocol. Serological examinations were performed in the central laboratory. Serological markers for HBV and HCV, including HBsAg, hepatitis B surface antibody (HBsAb), hepatitis B e antigen (HBeAg), hepatitis B e antibody (HBeAb), hepatitis B core antibody (HBcAb), and HCV antibody (HCVAb) were determined by enzyme-linked immunosorbent assays (ELISAs) (Kehua Bio-Engineering Co. Ltd., Shanghai, China) in accordance with the manufacturer’s instructions. Briefly, two negative controls, two positive controls, and one blank control were included in each plate. For HBV- or HCV-positive participants, repeated ELISAs were conducted (Abbott Microparticle Enzyme Immunoassay Method; Abbott Diagnostics Divisions, Santa Clara, CA, USA).

### Statistical analysis

The information obtained from the questionnaire form was entered into a database. The data were separated according to age and sex due to the complex sampling design of the study as well as according to response and analyzed using SPSS (SPSS, Inc., Chicago, IL, USA). Prevalence rates and constituent ratios as well as their 95% confidence intervals (CIs) for multi-morbidity were calculated.

## Results

### Epidemiological features of HBV and HCV infections

A total of 6642 participants were recruited for this study, and 101 were excluded from the final analysis due to the lack of complete questionnaire information or medical examination. The other 6541 participants responded to the questionnaire, for a response rate of 98.5%. Tests for sero-markers for HBV and HCV infections revealed that 127 cases were positive for HBsAg (1.9%), 3863 positive for HBsAb (59.1%), 4158 positive for HBcAb (63.6%), 28 positive for HBeAg (0.4%), 721 positive for HBeAb (11.0%), and 20 (0.3%) positive for HCVAb. After age-adjustment to represent the Changchun population, the weighted prevalence rates were: HBsAg (3.8%); HBsAb (55.2%); HBcAb (60.9%); HBeAg (0.72%); HBeAb (14.9%); and HCVAb (0.36%). Age was found as a major factor for the differences between unweighted and weighted prevalence rates. As shown in Table 1, the prevalence rates of HBsAg varied markedly among the study participants in the different age groups. The highest HBsAg-positivity rate found among the study subjects aged 40–49 years, whereas those individuals aged 50–59 years showed the highest prevalence rate of anti-HCV-positive results. The prevalence trend for HBcAb was similar to that for HBsAg, with the highest rate occurring among participants aged 50–59 years. The prevalence trend for HBsAb was different from that for HBsAg, showing a decrease according to advancing age from 84.8% (age < 1 year) to 49.0% (age 50–59 years). Furthermore, gender differences in the HBsAg- and anti-HCV positivity rates were observed, with higher rates found among males compared with females.

**Table 1:** General characteristics of study participants (adjusted)

Characteristic		Total	HBsAg(+)	HBsAb(+)	HBeAg(+)	HBeAb(+)	HBcAb(+)	Anti-HCV
		Population	%(95%CI)	%(95%CI)	%(95%CI)	%(95%CI)	%(95%CI)	%(95%CI)
Total number	Adjusted rate	6541	3.8(3.33, 4.26)	55.2(54.0, 56.4)	0.72(0.52, 0.92)	14.9(14.0.6, 15.8)	60.9(59.7, 62.1)	0.36(0.21, 0.50)
Gender								
	Male	3281	4.58(3.86, 5.30)	55.4(53.7, 57.1)	0.92(0.59, 1.25)	14.9(13.68, 16.12)	59.8(58.1, 61.5)	0.43(0.12, 0.65)
	Female	3260	3.0(2.41, 3.59)	54.9(53.2, 56.6)	0.51(0.27, 0.75)	14.8(13.62, 16.06)	61.9(60.3, 63.6)	0.33(0.13, 0.53)
Age (years)								
	< 1	382	0.0(0, 0)	84.8(81.2, 88.4)	0.0(0, 0)	8.9(6.0, 11.8)	75.1(70.8, 79.4)	0(0,0)
	1–4	1614	0.2(0, 0.42)	60.7(58.3, 63.1)	0.1(0, 0.25)	8.6(7.2, 10.0)	71.7(69.5, 73.9)	0.3(0.03, 0.57)
	5–14	1413	0.2(0, 0.43)	53.0(50.4, 55.6)	0.1(0, 0.26)	6.3(5.0, 7.6)	60.7(58.2, 63.2)	0.1(0, 0.26)
	15–29	1808	2.9(2.1, 3.7)	62.9(60.7, 65.1)	0.7(0.32, 1.10)	12.3(10.8, 13.8)	57.5 (55.2, 59.8)	0.4(0.1, 0.7)
	30–39	502	4.4(2.6,6.2)	53.6(49.2,58.0)	1.2 (0.25, 2.2)	14.5(11.4,17.6)	59.6(55.3,63.9)	0(0,0)
	40–49	455	5.9(3.73,8.07)	49.5(44.9,54.1)	0.9(0.03,1.77)	17.4(13.9,20.9)	62.9(58.5,67.3)	0.2(0,0.61)
	50–59	367	4.9(2.7,7.1)	49.0(43.9,54.1)	0.5(0,1.2)	23.4(19.1,27.7)	63.2(58.3,68.1)	1.1(0.03, 2.2)
HBV vaccination								
	Yes	4289	37(0.9)	2681(62.5)	13(0.3)	361(8.4)	2846(66.4)	9(0.2)
	No	961	42(4.4)	468(48.7)	7(0.7)	169(17.6)	544(56.6)	2(0.2)
	Unclear	1291	48(3.7)	714(55.3)	8(0.6)	191(14.8)	768(59.5)	9(0.7)

### Effects of hepatitis B vaccination on the HBsAg-positivity rates in all age groups

**Fig. 1:**
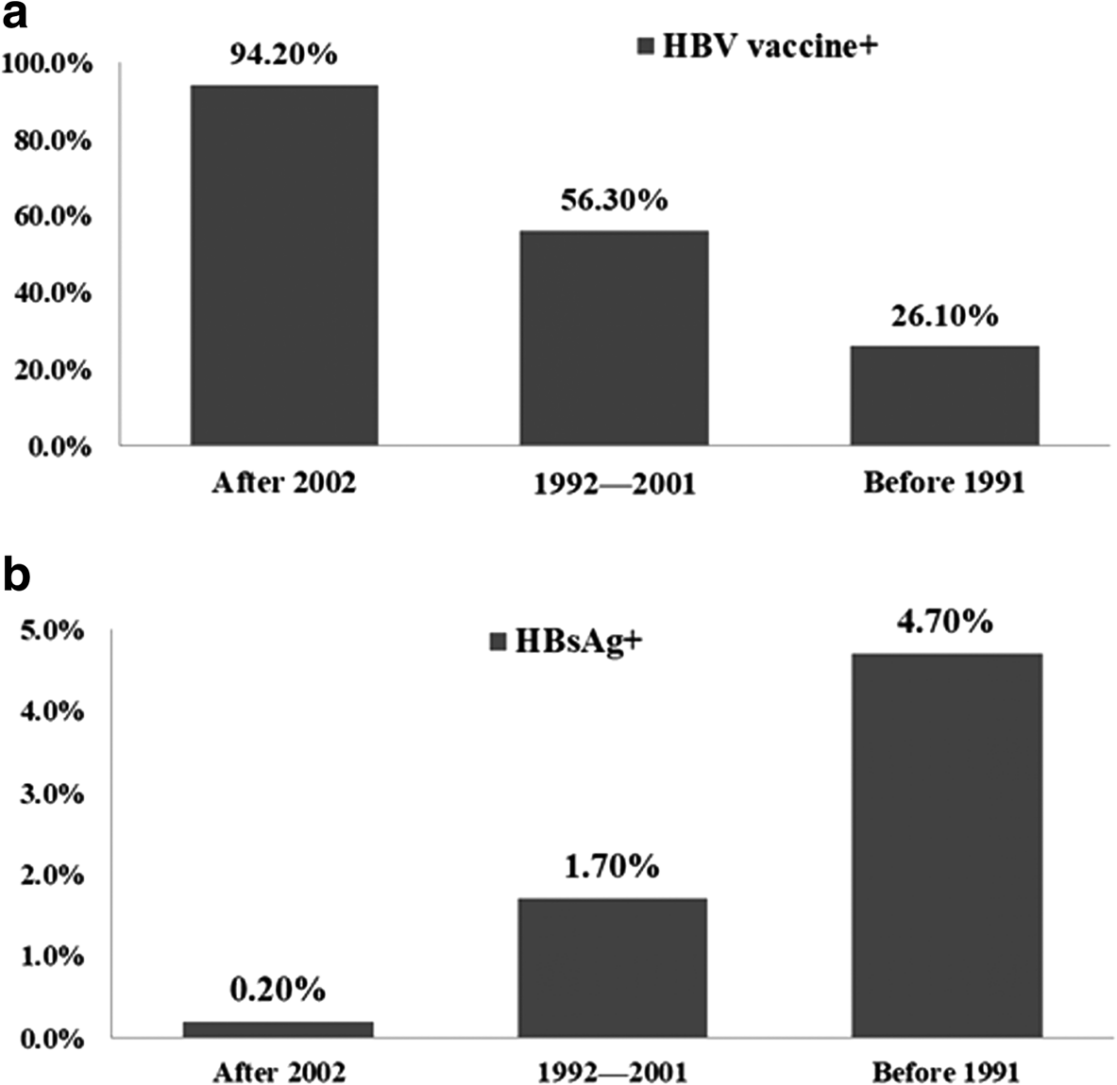
Effects of receiving hepatitis B vaccine on the HBsAg-positive rates in all age-groups (**a**) The coverage rates of the hepatitis B vaccine in all age-groups in this study; (**b**) Relationship between ages and the HBsAb-positive rates in all-age groups in this study

With the Chinese government implementing the policy of planned hepatitis B vaccine immunization nationwide and strengthening the blood management system in 2002, we anticipated that receiving the hepatitis B vaccine would have a beneficial impact on the HBsAg-positivity rates in the study participants. As expected, children who were born after the successful implementation of the public policy exhibited the highest coverage rate by the hepatitis B vaccine (94.2%) (Fig. 1a). Notably, children aged 0–14 years had a significantly lower HBsAg-positivity rate (0.2%) than individuals in any of the remaining age groups (Fig. 1b).

### Comparison of present and past HBsAg-positivity rates in China

**Fig. 2:**
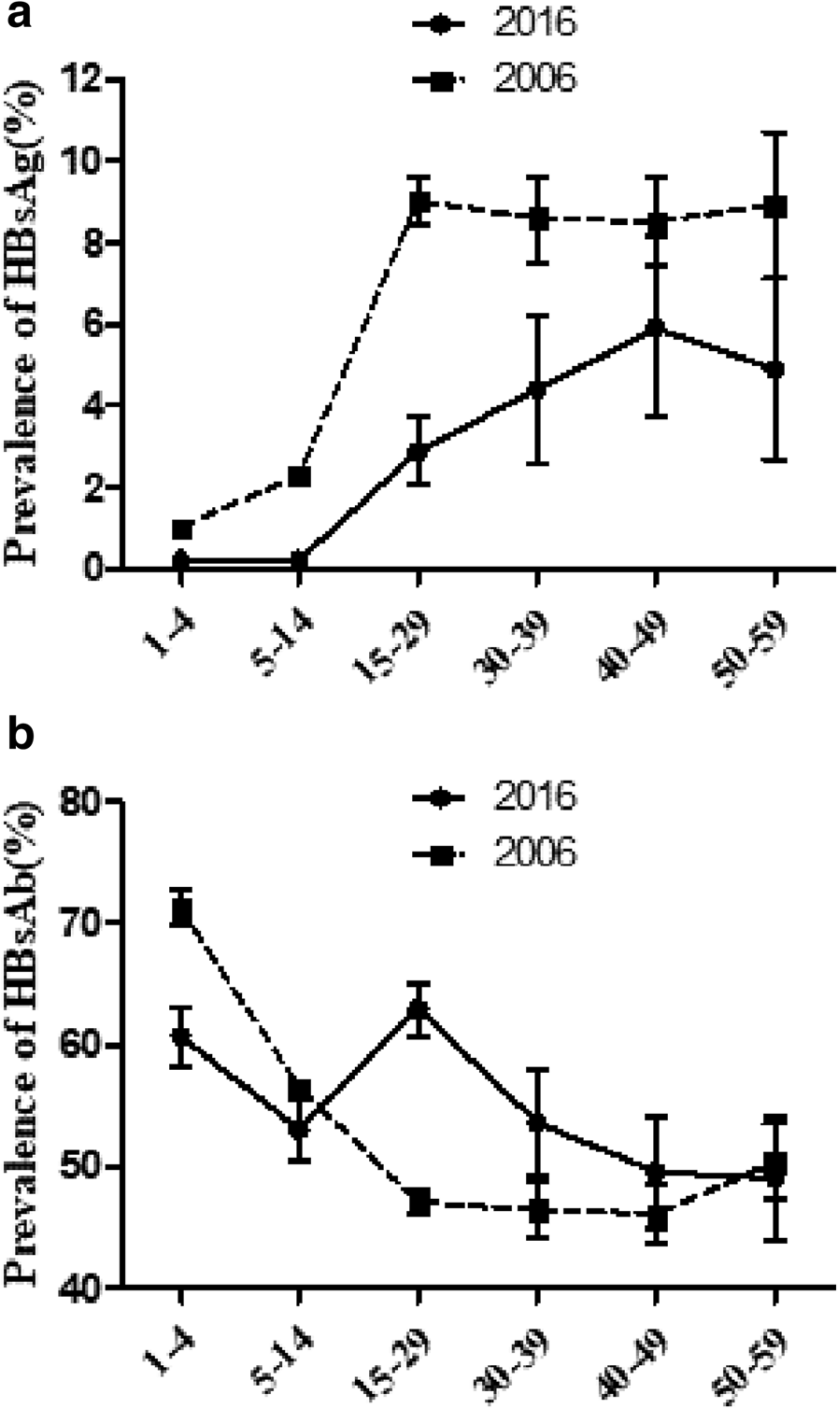
Comparison of the HBsAg-positive rates (**a**) and HBsAb-positive rate (**b**) of this study with those in the 2006 epidemiological serosurvey of hepatitis B in China

Next, we compared the HBsAg-positivity rates obtained in this study with those reported in the epidemiological serosurvey of hepatitis B conducted in 2006 [4]. Similar prevalence rates of HBsAg were found among children aged 1–4 years in both studies. However, for the other age groups (aged > 5 years), the HBsAg-positivity rates in our serosurvey were lower than those in the 2006 national survey (Fig. 2a). In comparison with the previous epidemiological serosurvey of hepatitis B in 2006 [4], we observed in the present study that the HBsAb-positivity rates were slightly higher in study participants aged > 15 years, but lower in children aged 0–14 years (Fig. 2b).

### Self-awareness of chronic HBV and basic knowledge about hepatitis B prevention

Our study also developed a questionnaire about basic HBV knowledge and conducted a survey among these participants. Overall, 67.2% of the participants realized that vaccination with the hepatitis B vaccine can prevent HBV infection and 38.9% of participants know that safe blood donation and blood transfusion are valid for impeding HBV transmission. However only 32.9% of the participants knew that teeth extraction and endoscopy should be carried out in a formal hospital. At the same time, 27.6% of participants did not know how to prevent hepatitis B. Our results also showed that only 16.5% of the HBsAg-positive population previously knew that they were positive for HBsAg (Table 2).

**Table 2:** Proportion of correct responses to questions about hepatitis B preventation

Whether you had positive HBsAg in the past years	correct responses(%)	Unclear(%)
1. It is previously known that I had positive HBsAg (HBsAg positive population)	21(16.5)	106(83.5)
Do you know how to prevent hepatitis B (all participants)		
1. Vaccination with hepatitis B vaccine can prevent HBV infection	4395(67.2)	2146(32.8)
2. Safe blood donation and blood transfusion	2539(38.9)	4002(61.2)
3. Teeth extraction, cosmetology and endoscopy are carried out in formal hospitals	2151(32.9)	4390(67.1)
4. I do not know	1808(27.6)	

## Discussion

“High” endemicity of chronic HBV infection refers to a positive rate of HBsAg ≥8%, while rates of 5–7%, 2–4%, and < 2% are classified as “high-intermediate”, “low intermediate”, and “low”, respectively [15]. China is among the countries with high endemicity with HBsAg-positivity rates varying by geography and subpopulation [16–18]. However, the results of HCV infection prevalence have been variegated in previous studies [19, 20]. The main findings of the present study are summarized as follows: (1) HBsAg was detected in 3.8% of the study participants, which corresponds to “low intermediate” endemicity and was markedly lower than the rate of 7.2% previously reported (2). Anti-HCV was detected in 0.36% of the current study participants, a rate that was lower than 0.43% as reported in the 2006 survey (3). For both HBsAg and anti-HCV, the positivity rates among male participants were significantly higher than those among female individuals. (4) Finally, children born after the vaccination strategy for hepatitis B was implemented had the lowest HBsAg-positivity rate of 0.2% among all age groups, indicating the beneficial effect of the public policy for the prevention and control of HBV infection in China.

In 1992, the WHO recommended that countries with a prevalence of high HBV infection burden administer the hepatitis B vaccine to children within their routine immunization schedule. Soon afterward, the Chinese government introduced the hepatitis B vaccine into routine vaccination programs nationwide. Furthermore, in 2002, the HBV vaccination was integrated into the Expanded Program on Immunization, and a free hepatitis B vaccine was available to all neonates, with families required to pay only a small service fee [21]. In 2005, the Chinese government began providing completely no-cost HBV vaccination for all newborn infants at no charge to their families. Due to the above effects, the coverage rates of HBV vaccination applied in three full doses within a timely manner increased markedly from approximately 30% in 1992 to as high as 90% in 2005 [22]. Moreover, the percentage of the population that had received the HBV vaccine increased from 26.2 to 56.4% over this time frame, suggesting that the implementation of the HBV vaccination policy in China has contributed to the prevention and control of HBV infection. In parallel, with an increase in the coverage rates of HBV vaccination in infants, the HBsAg-positivity rate dramatically declined, from 3.8% in individuals who were born before 1992 to 1.0% in those born after 2002. These results support the fact that HBV vaccination has greatly contributed to the decline in the prevalence of HBsAg.

We also compared our results with those obtained in a nationwide survey in 2006. The anti-HCV positivity rate was 0.36% in our study, which was slightly lower than the rate of 0.43% obtained in the previous study in 2006 in mainland China. This subtle decrease in the anti-HCV-positivity rate in China has been mainly attributed to the implementation of more stringent precautions for preventing and controlling HCV transmission through blood or blood product transfusion. However, we could not exclude the possibility that HCV infection rates vary across different regions of China. In fact, the prevalence of HCV infection in the northern area (0.53%) was shown to be higher than that in southern area (0.29%), but the anti-HCV prevalence did not differ significantly across the eastern (0.37%), middle (0.67%), and western (0.31%) regions of China [10]. Changchun city is located in the Northeast area in China, and the prevalence of HBV infection in this study was similar to that in eastern China.

Although the overall prevalence rates for both HBV and HCV infections have declined compared with those in the 2006 national survey, people in the high-risk groups may still be at greater risk than those in the general population. For example, the pooled prevalence rates of chronic HBV and HCV infections among kidney dialysis patients were as high as 11.9% and 41.1%, respectively [23, 24]. Similarly, the overall rates of HBsAg-positivity and anti-HCV-positivity were considerably greater in the following particularly high-risk groups: HIV-positive patients, injection drug users using shared needles, and individuals with unprotected sexual exposures [25, 26]. Furthermore, the epidemic of HCV infection was the most severe in the following provinces/autonomous regions: Hubei, Yunnan, Guangxi, Hunan, Xinjiang [19], and Fuyu [27]. Therefore, the “true” prevalence of HCV infection in China may exceed 1% as previously reported [9].

In May 2016, the WHO adopted a global hepatitis strategy with the goal of eliminating viral hepatitis as a public health threat by 2030. The targets to be achieved are ambitious: 30% of chronic hepatitis cases diagnosed by 2020 and 90% of chronic hepatitis cases diagnosed by 2030 [28]. To achieve this goal, the World Hepatitis Alliance (WHA) launched the World Hepatitis Day campaign entitled “Find the Missing Millions” in 2018 [29]. Our result shows that only 16.5% of the HBsAg-positive population previously knew that they were positive for HBsAg. This result indicates that we should promote the WHO’s goal through encouraging people to be screened and/or become advocates in the quest to find the undiagnosed. Because of the transmission of HBV/HCV, basic knowledge about hepatitis B is conducive to the protection of non-infected people, in whom an understanding of hepatitis B is important. Prevention efforts are hampered by limited understanding of infection burden and models of transmission. Previous work in China has shown that awareness of HBV and HCV infections is increasing. Furthermore, better knowledge of the available antiviral treatments for hepatitis prevention is needed to further reduce hepatitis infection rates in China.

Our study has a few limitations. Occult hepatitis C is usually characterized by the absence of serum anti-HCV with persistent levels of HCV-RNA. In this study, we could not determine the rate of hepatitis C self-awareness because we did not test for HCV-RNA. Other limitations may include a short study period and relatively small sample size. Additional studies are needed to overcome the above-mentioned limitations in order to confirm our results.

## Conclusions

In conclusion, the results of the present study demonstrate that the HBsAg-positive rate decreased significantly after implementation of the public policy on hepatitis B vaccination in China, suggesting a beneficial impact of the policy on the control of HBV infection. However, the anti-HCV-positivity rate showed a subtle decrease, indicating that continuous effort is still needed to strengthen the programs for the prevention and control of viral hepatitis in China.

## Abbreviations

CI: Confidence interval
HBsAg: Hepatitis B surface antigen
HBV: Hepatitis B virus
HCC: Hepatocellular carcinoma
HCV: Hepatitis C virus
PPS: Probability proportional to size
WHO: World Health Organization
